# Low-dose CT scan screening for lung cancer: comparison of images and radiation doses between low-dose CT and follow-up standard diagnostic CT

**DOI:** 10.1186/2193-1801-2-393

**Published:** 2013-08-21

**Authors:** Koji Ono, Toru Hiraoka, Asami Ono, Eiji Komatsu, Takehiko Shigenaga, Hajime Takaki, Toru Maeda, Hiroyuki Ogusu, Shintaro Yoshida, Kiyoyasu Fukushima, Michiaki Kai

**Affiliations:** Faculty of Nursing at Higashigaoka, Tokyo Healthcare University, 2-5-1, Higashigaoka, Meguro, Tokyo, Japan; Department of Radiology, Oita Red Cross Hospital, Japanese Red Cross Society, 3-2-37 Chiyomachi, Oita-City, Japan; Department of Radiology, Oita Prefectural Hospital, 476-1 Bunyo, Oita-City, Japan; Department of Radiology, Nagasaki Genbaku Isahaya Hospital, Japanese Red Cross Society, 986-2 Keya Taramichiyo, Isahaya-City, Japan; Department of Health Sciences, Oita University of Nursing and Health Sciences, 2944-9 Megusuno, Oita-City, Japan

**Keywords:** Lung cancer, Screening CT, Llow-dose, WAZA-ARI, ImPACT

## Abstract

**Objectives:**

This study aim to compare image quality and radiation doses between low-dose CT and follow-up standard diagnostic CT for lung cancer screening.

**Methods:**

In a single medical institution, 19 subjects who had been screened for lung cancer by low-dose CT before going through follow-up standard diagnostic CT were randomly selected. Both CT image sets for all subjects were independently evaluated by five specialized physicians.

**Results:**

There were no significant differences between low-dose CT screening and follow-up standard diagnostic CT for lung cancer screening in all 11 criteria. The concordance rate for the diagnoses was approximately 80% (p < 0.001) for all categories. Agreement of the evaluation of all categories in the final diagnosis exceeded 94% (p < 0.001). Five physicians detecting and characterizing the pulmonary nodules did not recognized the difference between low-dose CT screening and follow-up standard diagnostic CT. With low-dose CT, the effective dose ranged between 1.3 and 3.4 mSv, whereas in the follow-up diagnostic CT, the effective dose ranged between 8.5 and 14.0 mSv.

**Conclusion:**

This study suggests that low-dose CT can be effectively used as a follow-up standard diagnostic CT in place of standard-dose CT in order to reduce the radiation dose.

## Introduction

Lung cancer is the most common cause of death due to cancer in Japan. In 2010, the lung cancer screening rate was 23%, and 50,395 males and 19,418 females died from lung cancer, which accounts for more than 1 in 17 deaths (Statistics and Information Department, Minister’s Secretariat, Ministry of Health, Labour and Welfare 2011). Lung cancer screening using computed tomography (CT) has been carried out in Japan and the U.S. since 1993 (Kaneko et al. 1996), (Henschke et al. 1999). Many previous reports have indicated that CT is a good screening tool for the detection of small lung carcinomas. A high survival rate has been reported for cases of lung cancer detected with CT compared to chest radiography (Diederich et al. 2004), (Jett 2005), (Libby et al. 2006), (Henschke et al. 2006)-(Toyoda et al. 2008), (Fujikawa et al. 2008). However, it has been reported that low-dose CT screening for lung cancer may not significantly reduce the risk of advanced lung cancer or death from lung cancer (Bach et al. 2007). In November 2010, the National Lung Screening Trial (NLST) reported from initial trial results in the U.S. that 20% fewer lung cancer deaths were seen in trial participants that were screened with low-dose helical CT compared to those who were screened with chest X-rays (National Lung Screening Research Team 2011). The results of this trial provided direct evidence of the benefits of low-dose helical CT screening in an older, high-risk population with the habit of cigarette smoking. Issues discussed concerning low-dose CT include the high dose delivered per examination, cost, radiation exposure and image quality. However, low-dose CT screening may benefit individual at an increased risk for lung cancer, but uncertainly exists about the potential of screening and the generalizability of results (Bach et al. 2012).

Current studies have shown that not all pulmonary nodules detected by low-dose CT for lung cancer screening are malignant, and low-dose CT results in over-diagnosis caused by false-positive detection (Swensen et al. 2002), (Croswell et al. 2010). A previous report proposed that CT screening for lung cancer should be performed as a baseline. If detection of non-calcified nodules smaller than 5.0 mm in diameter is possible, a repeated annual screening, and not immediate work-up, would be justified in order to determine whether interim growth has occurred (Hensckle et al. 2004). On the other hand, previous reports have indicated that thin-section CT (TSCT) and high-resolution diagnostic CT may be helpful in differentiating small malignant nodules from benign nodules (Li et al. 2004), (Nakashima et al. 2006). Since small nodules are very difficult to identify, periodic follow-up examinations are required for diagnosis that take into account tumor doubling time (Aoki et al. 2000). Over 70% of participants had at least one follow-up CT. Non-calcified nodules are common among CT-screened high-risk subjects and can often be managed conservatively (Greenberg et al. 2012).

According to a questionnaire conducted by the Japanese Society of Medical Checkups, low-dose CT screening was conducted in only 30% institute in 2008 (Takizawa 2009). This may be because low-dose CT is generally recognized to create low quality images. The aim of this study was to compare images and radiation doses between low-dose for lung cancer screening and follow-up standard diagnostic CT in order to evaluate the efficacy of low-dose CT for lung cancer screening compared to repeated follow-up CT examinations.

## Materials and methods

### Design

In the present study, a database of low-dose CT images acquired for lung cancer screening and follow-up standard diagnostic CT images in the same patients were obtained from a single hospital. Diagnoses of lung cancer resulting from baseline low-dose CT screening and follow-up standard diagnostic CT are shown in Table 1. Results from the present study including two-year follow-up data are shown. The first nodule included was from May 2008 and the last was from August 2010. A total of 19 subjects who were examined with low-dose CT for lung cancer screening before going through follow-up standard diagnostic CT were randomly selected within 4 months of baseline screening. The 19 subjects (mean age, 60.6 years; age range 45–82 years) included a total of 9 men (mean age, 61.9 years; age range, 45–82 years) and 10 women (mean age, 58.8 years; age range, 49–66 years). Among the 19 subjects, 11 nodules were found in 1 subject, four nodules were found in 2 subjects, three nodules were found in 1 subject, and seven nodules were found in 1 subject.

**Table 1:** **Lung cancer diagnoses from baseline low-dose CT screening and follow-up standard diagnostic CT**

Subject number	Age	Sex	Number of pulmonary nodules	Days after diagnosis	Nodule diameter by low-dose CT (mm)	Nodule diameter by follow-up diagnostic CT (mm)	Diagnosis
1	57	M	1	43	15	15	Malignant nodule (adenocarcinoma as pure GGO with multiloculated cystic lesions )
2	46	M	1	15	13	13	Malignant nodule (pleural dissemination of adenocarcinoma as solid nodule with spiculation)
3	60	F	2	48	15 – 16	15 – 16	Two malignant nodules (bronchiolalveolar carcinomaı
4	66	F	7	3	4 – 7	4 – 7	All benign nodules
5	70	M	3	25	6	6	All benign nodules
6	60	M	4	38	3 – 8	3 – 8	Benign nodules
7	61	F	1	41	6	6	Benign nodule
8	59	F	4	27	4 – 7	4 – 8	All benign nodules
9	82	M	2	44	14 – 17	13 – 16	Possibly benign nodule, benign nodules
10	53	F	1	40	12	10	Benign nodule
11	65	F	1	35	11	10	Possibly benign nodule
12	49	F	1	74	6	5	Benign nodule
13	77	M	1	120	9	8	Benign nodule
14	60	M	2	119	4 – 7	4 – 8	All benign nodules
15	59	F	2	120	4 – 7	4 – 6	All benign nodules
16	61	F	1	118	7	7	Benign nodule
17	45	M	1	117	6	8	Benign nodule
18	64	M	1	120	9	8	Benign nodule
19	57	F	1	59	9	9	Possibly benign nodule

Image sets from the low-dose CT and follow-up CT of all 19 subjects were evaluated by five specialized physicians using 11 criteria related to malignancy that were developed for this study. All images were assessed independently by four radiologists with 31, 27, 21, and 8 years of experience in general radiology and a pulmonologist with 25 years of experience. Each image was ranked according to four-point confidence scales. Images were presented on a LSC 1 M monitor (EIZO Co. Ltd., Ishikawa, Japan) at a width and level of 1600 HU and −600 HU, respectively. The scales consisted of four sizes used to classify the lesions (20 ≤ S, 10 ≤ S < 20, 5 ≤ S < 10, S < 5), and four patterns of typical tumor appearance (solid nodule, mixed ground-glass opacity (GGO) or solid nodule and GGO, pure GGO, or cavitary). A total of four categories of calcifications were used (present, probably present, probably absent, absent), four categories of boundaries (irregular with spiculation, slightly irregular with spiculation, somewhat smooth, smooth), four categories of shapes (round, oval, polygonal, complex), four categories of margins (irregular with spiculation, somewhat irregular with slight spiculation, somewhat smooth, smooth), four categories of spiculations (present, probably present, probably absent, absent), four categories of glomerulus, four indrawn pleura signs, and four categories of air bronchogram (present, probably present, probably absent, absent). The combined diagnostic results of low-dose screening CT or follow-up standard diagnostic CT were then assigned a score as follows: 1, annual low-dose screening with CT is recommended; 2, suspicion of a noncancerous lesion and a follow-up CT in a hospital is recommended; 3, cancer or a noncancerous lesion is suspected; and 4, cancer is suspected.

Diagnosis concordance rates between low-dose CT and follow-up standard diagnostic CT were analyzed for 37 nodules. Diagnostic low-dose CT screening and follow-up standard diagnostic CT provided information concerning only nodules position. Correlation between low-dose CT for lung cancer screening and follow-up standard diagnostic CT of the lung was obtained by means of the chi-square test for independence.

### CT Techniques and procedures

All CT was performed on a MDCT scanner (16-slice Toshiba Medical Systems Activion) at a single institution. Low-dose CT screening was obtained with the following technical parameters: 120 kV peak, 30–50 mA, a detector thickness of 1 mm, an X-ray tube rotation speed of 0.75 s, 1 mm collimation × 16 mm, and a pitch factor of 1.438 mm within one breath-holding period. Reconstruction slice thickness was 3 mm and the slice interval was 3 mm. Follow-up standard diagnostic CT was obtained with the following technical parameters: 120 kV peak, AEC (max, 150 mAs) and detector thickness 1 mm, an X-ray tube rotation speed of 0.75 second, 1 mm collimation × 16 mm, and a pitch factor of 1.188 mm within one breath-holding period. Reconstruction slice thickness was 1 mm, and the slice interval was 1 mm with a CT reconstruction algorithm. Low-dose CT screening and follow-up standard diagnostic CT was performed with only a front view scanogram. The protocols used for scanning and reconstruction for the low-dose screening CT and follow-up standard diagnostic CT are shown in Table 2.

**Table 2:** **Scanning and reconstruction protocols of the toshiba MDCT scanner**

	Low-dose CT scan	Follow-up standard diagnostic CT scan
Number of detectors	16	16
kVp	120	120
mA	30–50	CT-AEC (SD = 70)
Seconds/rotation	0.75	0.75
mAs	22.5–37.5	Max 150
Pitch factor	1.438	1.188
Collimation	1 mm × 16	1 mm × 16
Reconstruction		
slice thickness (mm)	3	1
slice interval (mm)	3	1
Lung field	1600/-600	1600/-600
Mediastinal	400/35	400/35
Function	FC52	FC53

### Dose estimates

Tissue doses and effective doses of low-dose CT screening and follow-up standard diagnostic CT were estimated by the dose computational calculators, WAZA-ARI (Takahashi et al. 2011), (Ban et al. 2011a,b), ImPACT (Imaging Performance Assessment of CT scanners) (ImPACT: ctdi tables) and CT-EXPO v1.7.1 (Stamm & Nagel 2002). Dose calculation in WAZA-ARI utilizes the Japanese voxel phantom, where the Monte Carlo calculations are performed using the Particle and Heavy Ion Transport code System (PHITS).

## Results

Each of the five physicians analyzed 37 nodules in 19 subjects obtained by low-dose CT screening and follow-up standard diagnostic CT. Diagnosis of lung cancer resulting from baseline low-dose CT screening and follow-up standard diagnostic CT is shown in Table 3. There were no significant differences between low-dose CT and follow-upstandard diagnostic CT for lung cancer screening in all 11 criteria. The concordance rate for the diagnoses was approximately 80% (p < 0.001) for all categories. Agreement of the evaluation of all categories in the final diagnosis exceeded 94% (p < 0.001). Of the 19 subjects evaluated (9 men and 10 women; mean age, 60.6 years) there were 3 (2 men and 1 women; mean age, 54.3 years) malignant nodules and 16 (7 men and 9 women; mean age, 61.8 years) possibly benign nodules. The size of possibly benign nodules was 3 to 16 mm, and that of malignant nodules was 13 to 16 mm. The mean size of the four malignant nodules (14.6 mm) was larger than that of the 33 possibly benign nodules (6.8 mm).

**Table 3:** Lesion concordance rates between low-dose CT screening versus a follow-up standard diagnostic CT

	Observers						
Category	A	B	C	D	E	Average with lesion	Statistically significant difference
Size of nodule (mm)	100%	100%	100%	100%	100%	100%	p < 0.001
Pattern	92%	84%	78%	73%	84%	82%	p < 0.001
Calcification	100%	100%	100%	100%	95%	99%	p < 0.001
Boundary	95%	95%	76%	95%	84%	89%	p < 0.001
Shape	81%	97%	95%	95%	86%	91%	p < 0.001
Margin	89%	89%	92%	89%	95%	91%	p < 0.001
Spiculation	84%	95%	97%	76%	97%	90%	p < 0.001
Glomerulus	97%	95%	100%	92%	84%	94%	p < 0.001
Indrawn pleura sign	92%	84%	95%	73%	89%	87%	p < 0.001
Air bronchogram	86%	73%	84%	100%	70%	83%	p < 0.001
Final diagnosis	97%	97%	95%	84%	95%	94%	p < 0.001

**Figure 1:**
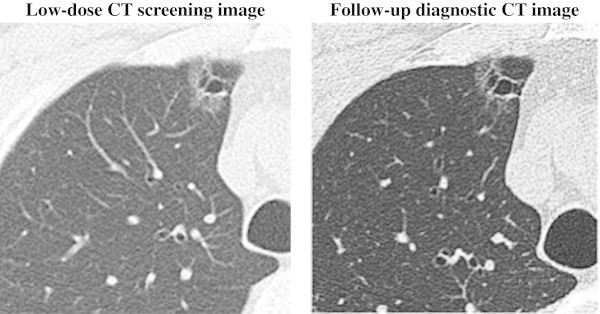
**Low-dose CT screening image and follow-up diagnostic CT image.** Low-dose CT screening image with an image slice thickness and interval of 3 mm. Follow-up diagnostic CT image with an image slice thickness and interval of 1 mm. 57-year-old man with adenocarcinoma presenting as pure GGO with multiloculated cystic lesions in the right upper lobe. Result of pathological staging operable patient (T1N0M0).

**Figure 2:**
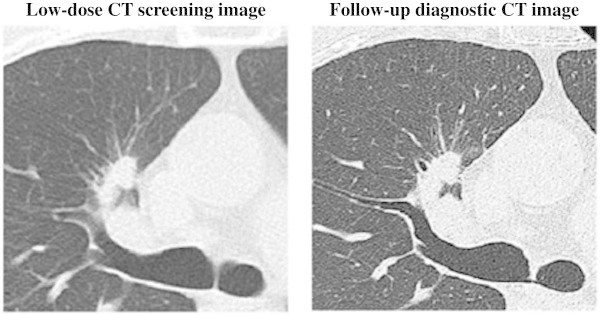
**Low-dose CT screening image and follow-up diagnostic CT image.** Low-dose CT screening image with an image slice thickness and interval of 3 mm. Follow-up diagnostic CT image with an image slice thickness and interval of 1 mm. A 46-year-old man with pleural dissemination and adenocarcinoma presenting as a solid nodule with spiculation in the right upper lobe. Results of pathological staging in an operable patient (T4N2M1).

**Figure 3:**
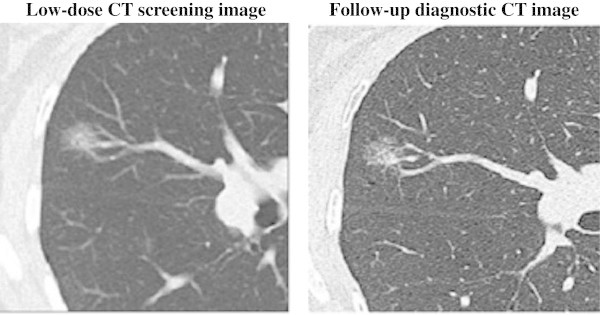
**Low-dose CT screening image and follow-up diagnostic CT image.** Low-dose CT screening image with a slice thickness and interval of 3 mm. Follow-up diagnostic CT image with an image slice thickness and interval of 1 mm. A 60-year-old woman with bronchioalveolar carcinoma (adenocarcinoma in situ) with mixed ground-glass opacity or pure ground-glass opacity in the right middle lobe (T1N0M0).

**Figure 4:**
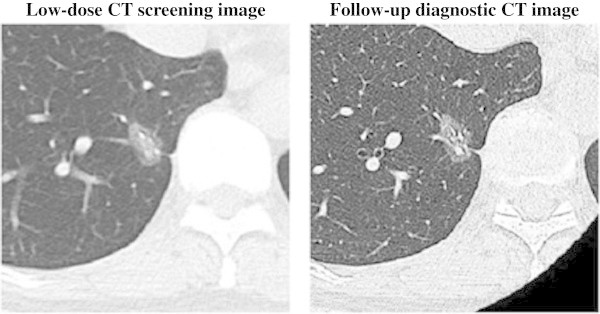
**Low-dose CT screening image and follow-up diagnostic CT image.** Low-dose CT screening image with a slice thickness and interval of 3 mm. Follow-up diagnostic CT image with an image slice thickness and interval of 1 mm. A 60-year-old woman with bronchioalveolar carcinoma (adenocarcinoma in situ) as mixed ground-glass opacity or pure ground-glass opacity in the right lower lobe (T1N0M0).

Evaluation concerning the nodules diagnosed as malignant accorded with the final diagnosis in the subjects. All physicians diagnosed 4 nodules in 3 subjects as malignant or suspected malignancy during follow-up examination. The four malignant nodules included an adenocarcinoma with cavitary lung lesion, pleural dissemination of an adenocarcinoma, and two bronchioloalveolar carcinomas, as shown in Figures 1, 2, 3 and 4. Two patient (3 nodules) diagnoses were confirmed at surgery, and 1 patient was diagnosed with inoperable lung cancer.

Dose to the internal organs under auto exposure control (AEC) was calculated by WAZA-ARI using the tube current obtained by follow-up standard diagnostic CT clinical data using the Toshiba Aquilion 16-MDCT (instead of the Toshiba Activity 16-MDCT). Radiation doses produced by lung CT with WAZA-ARI, ImPACT and CT-Expo indicated CTDIvol from the 16-slice Toshiba Medical System Activion are shown in Table 4. The average dose-length product (DLP) was 59.8 mGy × cm for low-dose TSCT using 30 mA for clinical data in 2 men and 2 women. The average DLP was 99.7 mGy × cm for low-dose CT using 50 mA in 7 men and 8 women. With low-dose TSCT, the doses calculated by WAZA-ARI ranged between 2.7 to 5.2 mGy in the lung with an 1.5 to 2.8 mSv effective dose, and with a follow-up standard diagnostic CT calculated dose of 15.1 to 25.2 mGy in lung with an 9.4. to 13.8 mSv effective dose (ICRP 103). With the low-dose CT using ImPACT, the dose was 5.8 mGy in the lung with a 3.4 mSv effective dose (ICRP 130), but with the follow-up standard diagnostic CT, it was 23 mGy in lung with a 14.0 mSv effective dose (ICRP 103). Using CT-EXPO, the dose was 5.3 mGy with a 2.8 to 3.3-mSv effective dose (ICRP 60) with the low-dose CT, while the lung dose ranged between 21 and 21.2 mGy and the effective dose between 11 and 13 mSv (ICRP 60) with a follow-up standard diagnostic CT.

**Table 4:** **Estimated radiation dose of lung CT and CTDIvol with the 16-slice Toshiba medical systems aquilion**

CT Scans	Low-dose CT scan				
CT dose soft	WAZA-ARI		ImPACT	CT-EXPO	
Auto exposure control	Without AEC		Without AEC	Without AEC	
Sex	Male	Female	Average adult	Male	Female
mAs	22.5 – 37.5	22.5 - 37.5	37.5	37.5	37.5
Lung (mGy)	2.7 – 4.6	3.1 – 5.2	5.8	5.3	5.3
Liver (mGy)	2.7 – 4.5	2.8 – 4.7	4.8	4.7	4.8
Breast (mGy)	2.1 – 3.5	2.2 – 3.6	4.3	5.4	5.4
ICRP103 (mSv)	1.7 – 2.8	1.5 – 2.5	3.4	-	-
ICRP60 (mSv)	1.5 – 2.5	1.3 – 2.2	2.9	2.8	3.3
DLP (mGy cm)	84 – 140	71 – 119	168	203	203
CTDIvol (mGy)	2.1 – 3.5	2.1 – 3.5	3.5	3.3	3.3
Average of DLP indicated value of 16 Toshiba rows CT (mGy x cm)	59.8 (n = 4) – 99.7(n = 15)				
CT Scans	Follow–up standard diagnostic CT scan				
CT dose soft	WAZA–ARI		ImPACT	CT–EXPO	
Auto exposure control	With AEC		Without AEC	Without AEC	
Sex	Male	Female	Average adult	Male	Female
mAs	150 (Max)	150 (Max)	150	150	150
Lung (mGy)	15.1 – 22.1	17.9 – 25.2	23.0	21.0	21.2
Liver (mGy)	18.6 – 21.5	19.0 – 22.5	19.0	18.7	19.3
Breast (mGy)	10.4 – 17.0	11.6 – 17.5	17.0	21.4	21.4
ICRP103 (mSv)	11.1 – 13.8	9.4 – 12.1	14.0	-	-
ICRP60 (mSv)	10.3 – 12.5	8.5 – 10.6	12.0	11	13
DLP (mGy cm)	545 – 679	455 – 574	673	810	810
CTDIvol (mGy)	13.5 – 16.8	13.3 – 16.8	13.9	13.3	13.3
Average of DLP indicated value of 16 Toshiba rows CT (mGy x cm)	474.7 (n = 19)				

*These radiation transport calculations were performed using the JM phantom with WAZA-ARI, and the MIRD-5 type phantom with ImPACT and the GSF phantom with CT-Expo. The JM phantom was constructed from CT images of a healthy Japanese male adult. The height and the weight were 171 cm and 65 kg, respectively. The MIRD-5 type phantom was a hermaphroditic adult. For the MIRD–5 type phantom, the height and the weight were 174 cm and 70 kg, respectively. For the GSF-ADAM phantom, the height and the weight were 170 cm and 70 kg, respectively. For the GSF-EVA phantom, the height and the weight were 160 cm and 60 kg, respectively.

## Discussion

This is the most important finding in smaller nodules concerning improvement of survival. Many previous reports suggest the merit of early detection (Suzuki et al. 2009), (National Cancer Institute 2011). The problem is that low-dose CT for lung cancer screening creates an endemic of over-diagnosis through false-positives because of the high sensitivity and capacity for detection. The present study revealed that only a small number of low-dose CT scans used for lung cancer screening result in over-diagnosis compared to follow-up standard diagnostic CT. These features appear to be a specific characteristic of high-resolution CT.

According to the UNSCEAR (United Nations Scientific Committee on the Effects of Atomic Radiation) 2008 report, the effective dose of natural background radiation is 2.4 mSv (2010). A previous study reported that the background dose was 8.71 mGy in the lung, with an effective dose that ranged between 3.61 and 3.64 mSv (ICRP 60) using an anthropomorphic phantom, which is a model of a Japanese adult (RANDO; Alderson Research Laboratory, Salem, NY, USA, 163 cm height, 53 kg weight, without legs or arms) (Nishizawa et al. 1996). The scanning parameters for lung cancer used for a single spiral CT were 120 kVp and 50 mA, a slice thickness of 10 mm, tube rotation speed of 2 s, a reconstructed image pitch of 10 mm, and a table speed of 10 mm/s. The effective dose of the low-dose CT ranged between 1.6 and 3.4 mSv. Comparison of effective doses between 10-mm slice thickness and 1-mm slice thickness was the same using modern CT units. In digital and analog chest X-ray systems, the radiation dose was extremely low, with averages ranging between 73 and 198 μGy. However, a phantom study that simulated Noguchi’s Type A small adenocarcinoma revealed that the detection rate was very low by digital and analog chest X-ray systems (Ono et al. 2005).

The present study indicated that low-dose CT screening provided diagnoses similar to standard diagnostic CT. The image quality of low-dose CT may be high enough for it to be used for the diagnosis of lung cancer as a method of repeated follow up. We suggest that low-dose CT for lung cancer screening should be used as a follow-up to standard diagnostic CT in place of standard-dose CT. The dose to the lung in low-dose CT is approximately one-sixth that of standard CT. Furthermore, it is suggested that in a high-risk population with the habit of cigarette smoking, annual low-dose CT for lung cancer screening is modestly recommended. It is important to create an effective low-dose CT protocol for lung cancer screening that takes into consideration both radiation dose and image quality.

## Conclusion

Since small nodules in the lungs are very difficult to identify, periodic follow-up examinations are required for diagnosis of lung cancer. This study suggests that low-dose CT can be effectively used as a follow-up standard diagnostic CT in place of standard-dose CT in order to reduce the radiation dose.
